# Oral rehabilitation using three-dimensional-guided autotransplantation following pediatric rhabdomyosarcoma and secondary non-Hodgkin lymphoma with severe dental late effects: a case report

**DOI:** 10.1186/s13256-025-05813-y

**Published:** 2026-01-21

**Authors:** Miks Lejnieks, Zanna Kovaļova, Anda Slaidina, Ģirts Šalms, Sergio E. Uribe

**Affiliations:** 1 Department of Oral and Maxillofacial Surgery and Oral Medicine, Rīga Stradiņš University, Rīga, Latvia; 2 Department of Hemato-Oncology, Children’s Clinical University Hospital, Riga, Latvia; 3 Department of Pediatrics, Faculty of Medicine, Riga Stradins University, Riga, Latvia; 4 Department of Prosthodontics, Rīga Stradiņš University, Rīga, Latvia; 5 Institute of Stomatology, Rīga Stradiņš University, Rīga, Latvia; 6 Department of Conservative Dentistry and Oral Health, Rīga Stradiņš University, Rīga, 1007 Latvia; 7 Baltic Biomaterials Centre of Excellence, Headquarters at Rīga Technical University, Rīga, Latvia

**Keywords:** Tooth autotransplantation, Pediatric cancer, Rhabdomyosarcoma, Non-Hodgkin lymphoma, Dental anomalies, Micro-CT, 3D printing, Implant rehabilitation, Case report

## Abstract

**Background:**

Pediatric cancer therapies often cause severe dental developmental disturbances. While extractions are common, biological rehabilitation using autotransplantation is rarely reported, particularly in patients with secondary malignancies and complex medical histories. This case describes multidisciplinary oral rehabilitation using tooth autotransplantation, guided implant placement, and three-dimensional digital planning in a young patient with dual pediatric malignancies and severe dental anomalies.

**Case presentation:**

A 17-year-old White female patient from Latvia presented with multiple dental anomalies following intensive treatment for rhabdomyosarcoma at the age of 11 years and subsequent B-cell precursor non-Hodgkin lymphoma at the age of 14 years. Oncologic management included systemic chemotherapy, intrathecal therapy, and image-guided fractionated intensity-modulated radiation therapy to the entire brain and spinal cord (18 Gy total dose, 2 Gy per fraction, 9 fractions), resulting in severe root resorption of tooth 16, bilateral agenesis of mandibular second premolars (35, 45), and structural malformations of third molars. Treatment involved orthodontic space preparation, extraction of tooth 16, and autotransplantation of tooth 28 using a cone-beam computed tomography-guided three-dimensionally printed replica. Guided implant placement was performed in sites 35 and 45 after space opening. Extracted third molars were analyzed using micro-computed tomography, revealing therapy-induced disorganized dentin-pulp morphology and abnormal mineralization. At 3-year follow-up, the autotransplanted tooth exhibited complete root maturation and stable periodontal integration. Both implants showed stable osseointegration with no complications at 2-year follow-up. The patient reported high functional and aesthetic satisfaction.

**Conclusions:**

Biological rehabilitation using tooth autotransplantation and guided implant placement is feasible and effective in select patients post-oncology who have severe therapy-related dental anomalies. Integration of three-dimensional digital planning improves surgical accuracy and treatment outcomes. Interdisciplinary care is essential for managing complex oral rehabilitation after pediatric cancer therapy.

## Background

Pediatric oncology advances have improved survival rates. However, chemotherapy and radiotherapy cause long-term complications in developing tissues, particularly teeth [1]. Odontogenesis is a complex process susceptible to systemic treatments [2]. Anti-cancer therapy during critical dental formation stages causes permanent abnormalities, including tooth agenesis, malformed roots, enamel defects, delayed eruption, and altered dentin and pulp structures [3, 4].

Literature describes various dental late effects in patients who underwent cancer treatment [5]. Treatment of dental complications in cancer patients often focuses on extractions and subsequent rehabilitation [6]. However, biological and conservative approaches, such as autotransplantation, remain rarely documented [3, 6]. These patients present anatomical and physiological limitations such as compromised bone quality, altered root morphology, and unpredictable periodontal conditions that complicate conventional dental rehabilitation [7, 8].

Current clinical guidelines and systematic reviews recommend extracting problematic teeth before cancer treatment to prevent complications [9, 10]. Pretreatment extraction prevents osteoradionecrosis (ORN) in patients receiving head and neck radiotherapy and life-threatening infections in patients undergoing chemotherapy or hematopoietic cell transplantation [10]. These recommendations focus on prevention through extraction before treatment begins. However, current literature provides limited guidance on biological rehabilitation options for patients who complete cancer therapy and achieve immune reconstitution. This case demonstrates that autotransplantation can succeed in patients post-oncology who have completed therapy and have stable immune function, offering a conservative and biological alternative to conventional prosthetic rehabilitation.

This case report presents long-term oral rehabilitation of a young female patient who underwent cancer treatment for rhabdomyosarcoma and stage IV B-cell precursor non-Hodgkin lymphoma (NHL). The patient received intensive multimodal therapy with systemic chemotherapy and craniospinal irradiation during mixed dentition. Resulting dental anomalies included bilateral agenesis of mandibular second premolars, severe root resorption of a maxillary first molar, and structural malformations of third molars. While these anomalies are consistent with therapy-related effects during odontogenesis, the bilateral agenesis of premolars may also reflect developmental variation, as congenital absence of second premolars occurs in approximately 3–4% of the general population [11]. We describe a multidisciplinary approach integrating autotransplantation, guided implant placement, and three-dimensional (3D) digital technologies. This is the first report combining dual malignancy survivorship, 3D-guided autotransplantation, implant rehabilitation, and micro-computed tomography(CT) characterization in a single pediatric cancer survivor. Previous reports have documented these components separately, but their integration with structural analysis of therapy-related dental damage has not been described. Extracted third molars were evaluated using micro-computed tomography to assess developmental disturbances linked to oncologic therapy. This case demonstrates the feasibility and clinical success of a biologic and conservative approach in a medically complex adolescent.

## Case presentation

This case is reported following the Case Report (CARE) guidelines [12]. The completed CARE checklist is provided as Supplementary File 1. Written informed consent for publication was obtained from the patient. This study was conducted in accordance with the Declaration of Helsinki and approved by the Rīga Stradiņš University Research Ethics Committee (Decision No. 6-1/08/12, 23 July 2020).

### Patient information

#### De-identified patient information

A 17-year-old Latvian White female presented to the Department of Oral and Maxillofacial Surgery at Riga Stradiņš University for treatment of dental anomalies.

#### Primary concerns and symptoms

The patient presented with functional and aesthetic challenges, particularly with chewing and spacing in the premolar regions. She experienced pain and discomfort during mastication, with specific concerns about the stability of tooth 16.

#### Medical, family, and psychosocial history

**Fig. 1:**
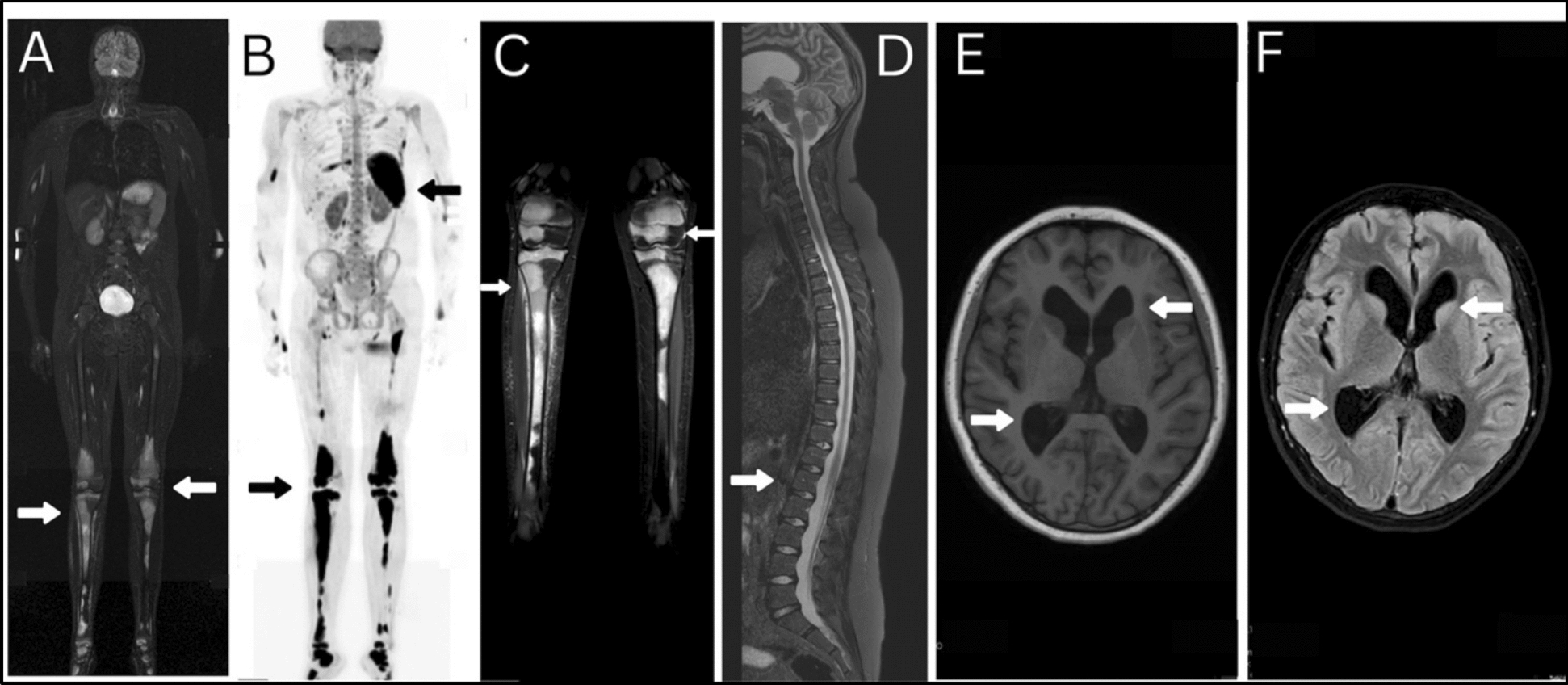
Magnetic resonance imaging, positron emission tomography, and central nervous system imaging in rhabdomyosarcoma with relapsed non-Hodgkin lymphoma. **A** (age 11 years) Whole-body coronal short tau inversion recovery magnetic resonance imaging showing diffuse bone marrow infiltration, particularly in the proximal and distal tibias (white arrows), consistent with metastatic involvement. **B** (age 11 years) Whole-body positron emission tomography scan reveals hypermetabolic activity (black arrows) in the right tibia, femur, and pelvis, confirming the presence of multiple skeletal metastases. **C** (age 11 years) Bilateral lower leg coronal magnetic resonance imaging T1 images, highlighting low signal intensity lesions in both tibias (white arrows), suggestive of tumor infiltration and correlating with positron emission tomography findings. **D** (age 11 years) Sagittal T2-weighted spinal magnetic resonance imaging demonstrates abnormal signal intensity and swelling in the thoracic spinal cord (white arrow), raising suspicion of leptomeningeal spread or spinal involvement. **E** (age 14 years) Axial T1-weighted magnetic resonance imaging shows ventriculomegaly and sulcal efacement, suggesting obstructive hydrocephalus due to central nervous system involvement of non-Hodgkin lymphoma. **F** (age 14 years) Axial fluid-attenuated inversion recovery magnetic resonance imaging reveals periventricular and basal ganglia hyperintensities, likely related to leukemic infiltration or treatment-induced leukoencephalopathy. White arrows in **E** and **F** showing where sarcoma and lymphoma were observed

The patient had a significant oncological history beginning in July 2015 at the age of 11 years, when she presented with slight swelling in the proximal part of her right lower leg. Initial biopsy suggested metastatic rhabdomyosarcoma involving bone and bone marrow. Whole-body magnetic resonance imaging (MRI) confirmed diffuse marrow infiltration in the right lower leg (Fig. 1 A–D). No primary tumor was identified.

Chemotherapy began on 28 October 2015 under the Cooperative Weichteilsarkom Studiengruppe (CWS)-2012 protocol for metastatic tumors. Histological reassessment at the Kiel Pediatric Pathology Registry revised the diagnosis to an undifferentiated small blue cell tumor, possibly lymphoma. This led to reclassification on 17 February 2016 as an undifferentiated small blue cell tumor in the right tibia with multiple bone metastases.

In January 2018, the patient was hospitalized with nocturnal headaches, nausea, and vomiting. Cerebrospinal fluid analysis confirmed central nervous system relapse with B-cell precursor variant of non-Hodgkin lymphoma (B-precursor acute lymphoblastic leukemia [B-ALL]). Immunophenotyping revealed a 71% blast population, and flow cytometry showed markers consistent with B-ALL (CD19+ , CD10+ , CD22+ , CD66c+). Molecular studies demonstrated clonal rearrangements in immunoglobulin heavy chain (IGH), TCRB, TCRG, and TCRD, confirming leukemic involvement.

Treatment included intensive systemic chemotherapy, intrathecal chemotherapy, and image-guided fractionated intensity-modulated radiation therapy (IMRT) to the entire brain and spinal cord (18 Gy total dose, 2 Gy per fraction, 9 fractions delivered from 17 September 2018 to 28 September 2018). She experienced complications, including veno-occlusive disease, obstructive hydrocephalus, coagulopathy, and febrile neutropenia (Fig. 1E, F). Maintenance therapy was completed in February 2020. Follow-up brain MRIs have shown no pathological findings since completion of treatment.

#### Relevant past interventions and outcomes

As a late effect of oncologic treatment, the patient developed multiple dental abnormalities, including severe root resorption of the maxillary first molar (tooth 16), bilateral agenesis of teeth 35 and 45, and developmental anomalies in third molars (18, 28, 38 and 48) with incomplete root formation and altered morphology.

### Clinical findings

Extraoral examination revealed no facial asymmetry, swelling, or lymphadenopathy. No signs of trismus, sinus tract, or soft tissue inflammation were present. The patient appeared healthy and in stable general condition.

Intraoral examination showed good oral hygiene and healthy gingival tissues. No active caries or signs of periodontal disease were observed. Tooth 16 exhibited an intact crown with deep probing depths on the buccal aspect (6 mm) and no mobility, but with percussion sensitivity. Occlusion was stable but asymmetric due to missing mandibular premolars.

Dental anomalies identified included: tooth 16 with advanced external root resorption and poor long-term prognosis; tooth 28 as an immature third molar with incomplete root formation, selected as donor for autotransplantation; teeth 35 and 45 with bilateral agenesis requiring orthodontically created space for implant placement; teeth 18 and 48 as retained third molars with developmental anomalies, later extracted for micro-CT analysis.

### Timeline

**Fig. 2:**
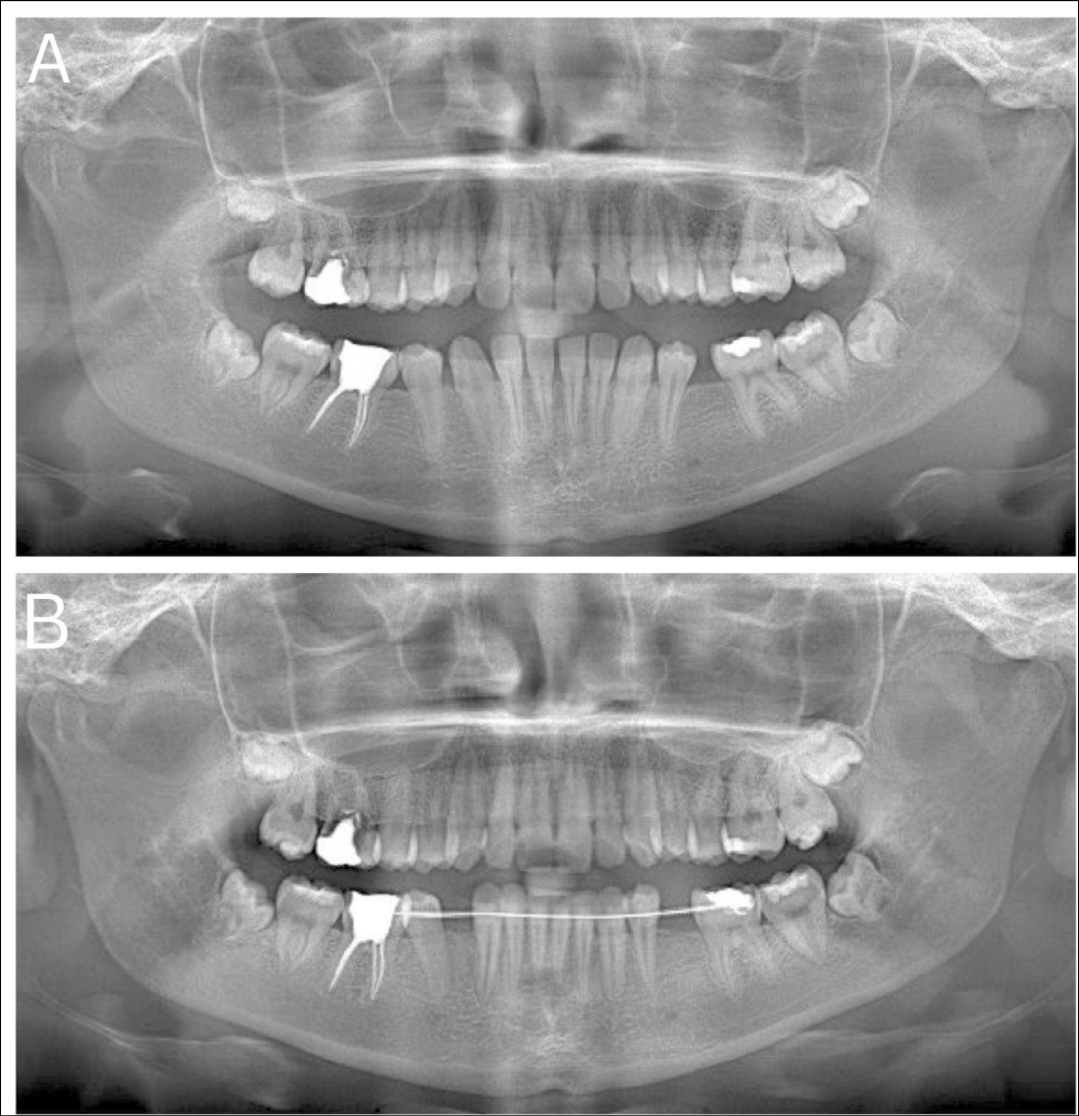
Orthopantomogram progression showing worsening root resorption of tooth 16 and delayed decision for autotransplantation. **A** (age 16 years) Panoramic radiograph taken 6 months after radiotherapy showing progressive root resorption of tooth 16. Initial consideration of autotransplantation was rejected at this stage. **B** (age 18 years) Follow-up panoramic radiograph taken 1 year later demonstrating further root resorption of tooth 16, with worsened structural integrity. Based on disease progression, autotransplantation was approved. Note the presence of orthodontic appliances initiating space preparation. Both images were taken before surgical intervention

In 2015, at the age of 11 years, the patient presented with swelling in the right lower leg, initially diagnosed as rhabdomyosarcoma and later reclassified as an undifferentiated small blue cell tumor with bone metastases. Central nervous system relapse occurred in 2018 at the age of 14 years and was diagnosed as B-cell precursor non-Hodgkin lymphoma following cerebrospinal fluid analysis. NHL treatment protocol was initiated in 2018, including chemotherapy, intrathecal therapy, and image-guided fractionated intensity-modulated radiation therapy (IMRT) to the entire brain and spinal cord (18 Gy total dose, 2 Gy per fraction, 9 fractions), with complications including veno-occlusive disease, obstructive hydrocephalus, and febrile neutropenia. Maintenance therapy was completed in February 2020. Follow-up brain MRIs have been performed every 6 months, with no new pathological findings, so remission was achieved in 2020 at the age of 16 years. In 2020, at the age of 16 years, autotransplantation was initially considered but rejected due to insufficient time since completion of radio/chemotherapy (only 6 months post-treatment) and immature developmental stage of the donor tooth. Severe root resorption of tooth 16 was diagnosed radiographically in 2021 (Fig. 2A).

**Fig. 3:**
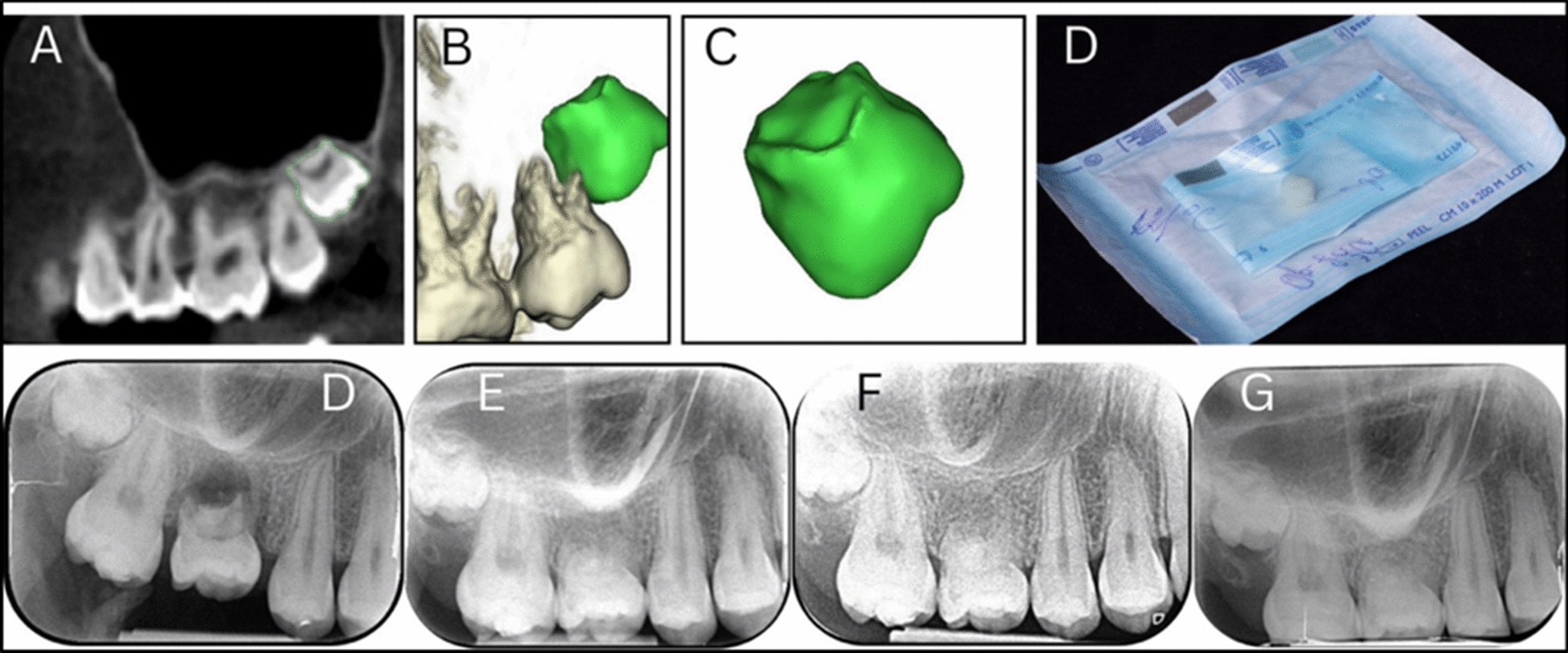
Planning and follow-up of autotransplantation of donor tooth 28 using three-dimensional technology. **A** Cone-beam computed tomography image showing the donor tooth 28 (outlined in green) selected for autotransplantation. **B**, **C** Three-dimensional reconstruction of tooth 28 with surrounding structures and an isolated digital model, used to design a surgical replica. **D** Sterilized three-dimensionally printed tooth replica packaged for surgical use, enabling trial fitting and minimizing extraoral donor handling. **E** Periapical radiograph taken immediately after autotransplantation into site 16. **F** Radiograph at 6-month follow-up, demonstrating early signs of periodontal healing and no signs of resorption or ankylosis. **G** Follow-up at 1 year showing continued root maturation and periodontal integrity. **H** Follow-up at 2 years confirming long-term success with stable position; no radiographic signs of pathology

**Fig. 4:**
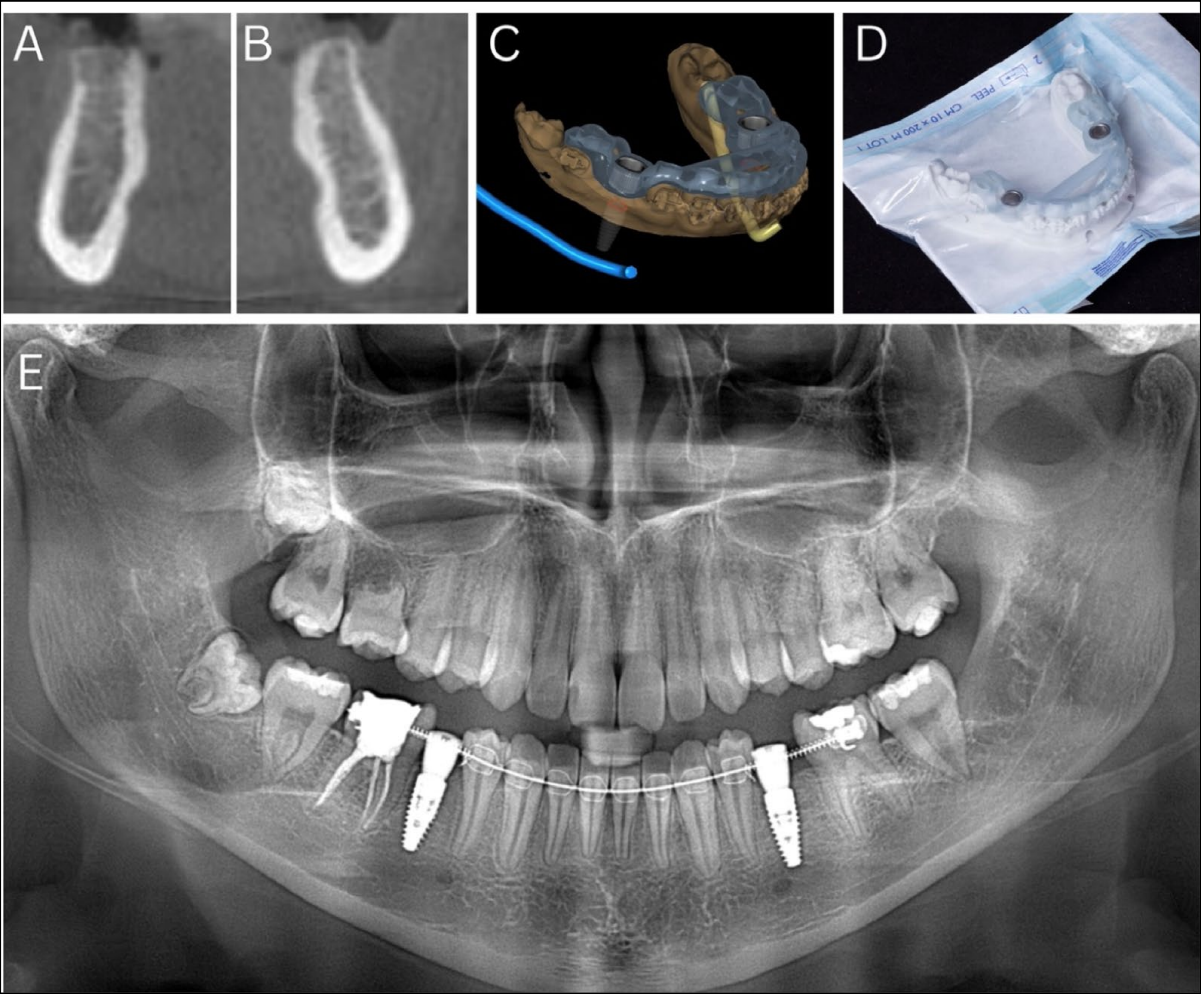
Digital planning and implant placement in agenesis sites using three-dimensionally printed surgical guide. **A**, **B** Cone-beam computed tomography cross-sectional images of mandibular sites 45 (**A**) and 35 (**B**), showing ridge dimensions following orthodontic space opening in areas of congenital agenesis of the second premolars. **C** Digital planning of implant positioning and surgical guide design based on cone-beam computed tomography and intraoral scan data. **D** Sterilized three-dimensionally printed surgical guide fabricated for use during implant placement. **E** Postoperative panoramic radiograph showing placement of Straumann bone level tapered implants (diameter 4.1 mm, length 10 mm) in sites 35 and 45 via guided flap surgery

**Fig. 5:**
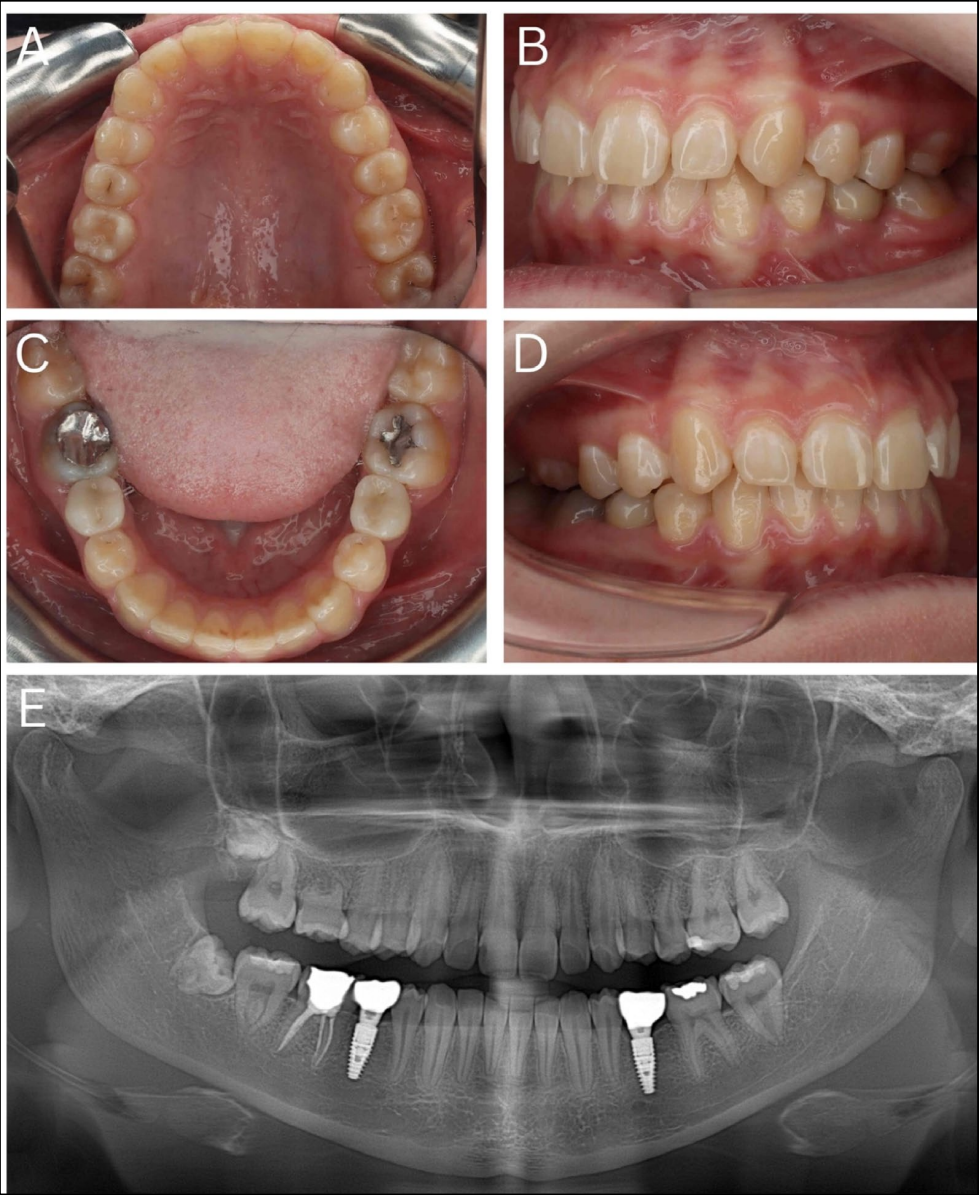
Clinical and radiographic follow-up 2 years after implantation and 3 years after autotransplantation. **A** Occlusal view of the maxillary arch demonstrating stable occlusion and integration of the autotransplanted tooth at site 16. **B**, **D** Right and left lateral intraoral views showing well-aligned occlusion and healthy gingival tissues around the autotransplanted and implant-supported restorations. **C** Occlusal view of the mandibular arch showing implant-supported crowns in regions 35 and 45, with healthy peri-implant tissues. **E** Panoramic radiograph taken 2 years after implantation and 3 years after autotransplantation showing stable implant osseointegration and transplanted tooth function; third molars (teeth 18 and 48) were later extracted for micro-computed tomography analysis of therapy-related changes

**Fig. 6:**
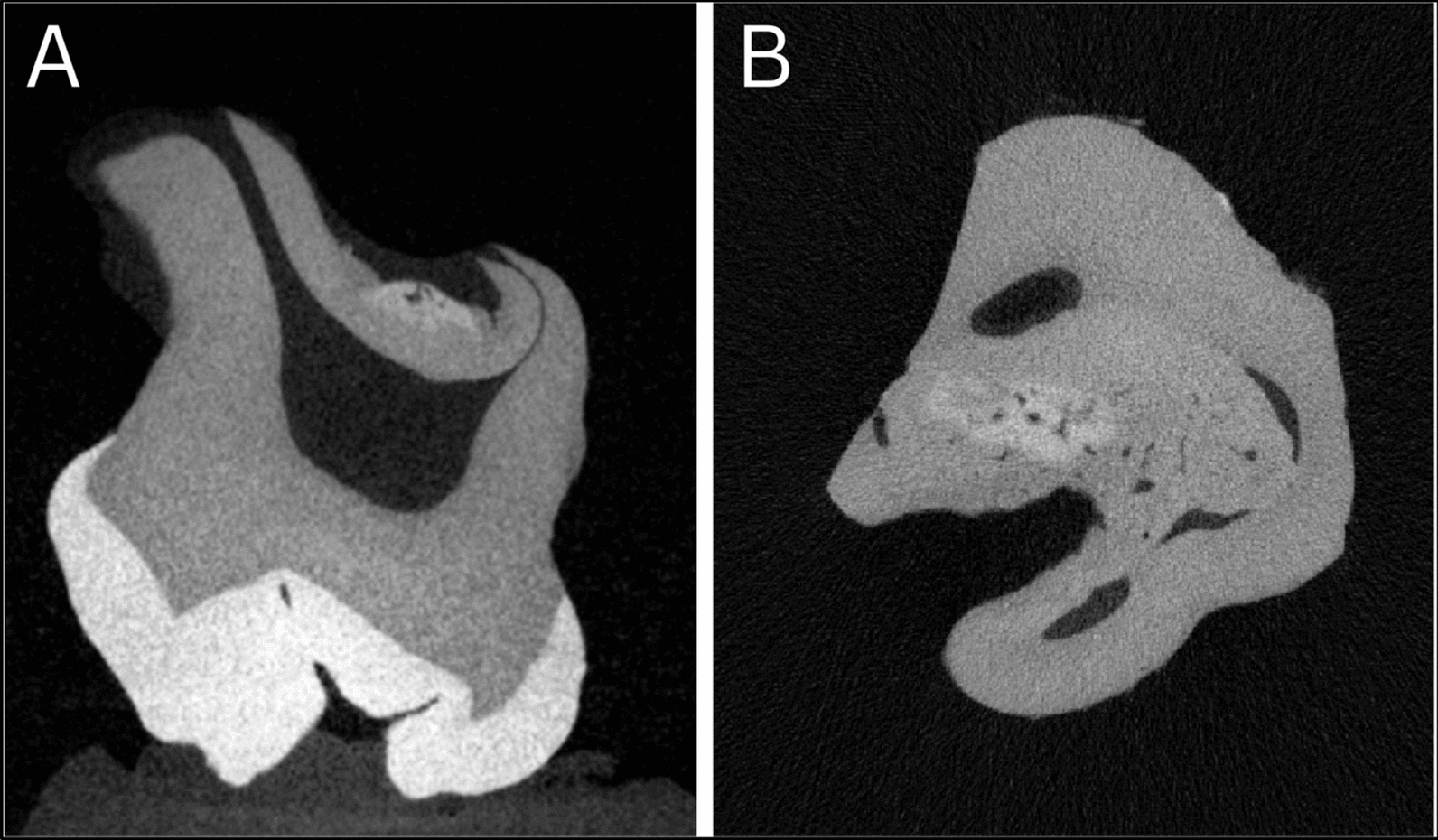
Micro-computed tomography imaging of extracted third molars using Rigaku CT lab HX130. **A** Micro-computed tomography image of tooth 18 showing irregular root canal morphology and thinning of dentinal walls, with evidence of altered dentin structure. **B** Micro-computed tomography image of tooth 48 revealing disrupted root architecture, abnormal pulp chamber formation, and areas of mineralization defects

Orthodontic treatment was initiated for space preparation in 2021 and completed in 2022 (Fig. 2B), followed by the extraction of tooth 16 and autotransplantation of tooth 28 using a 3D replica as a guide in 2021 (Fig. 3). Implants were placed in sites 35 and 45 using guided surgery in 2022 (Fig. 4). Subsequently, 3-year follow-up for the autotransplanted tooth and 2-year follow-up for the implants were documented in 2024 at the age of 20 years, confirming stable function and aesthetics (Fig. 5). Teeth 18 and 48 were extracted for micro-CT analysis using Rigaku CT Lab HX130 in 2025 at the age of 21 years (Fig. 6). A detailed summary of the chronological sequence of oncologic events, dental diagnoses, interventions, and outcomes is presented in Table 1.

**Table 1:** Clinical timeline

Years/age	Event	Diagnosis/intervention	Outcome
2015 (11 years)	Initial presentation	Right lower leg swelling; initially diagnosed as rhabdomyosarcoma but later reclassified as undifferentiated small blue cell tumor with bone metastases	Initiated CWS-2012 chemotherapy protocol
2018 (14 years)	CNS relapse	B-cell precursor non-Hodgkin lymphoma confirmed via CSF analysis	Started NHL protocol with systemic and intrathecal chemotherapy + IMRT to the entire brain and spinal cord (18 Gy total dose, 2 Gy per fraction, 9 fractions)
2020 (16 y)	Maintenance completed	Completion of multimodal oncologic therapy	Remission achieved; stable follow-up MRIs
2021 (17 years)	Autotransplantation consideration	Autotransplantation initially considered but rejected by team and patient	Insufficient time post-therapy (6 months); immature donor tooth
2021 (17 years)	Dental diagnosis	Severe root resorption of tooth 16 diagnosed radiographically	Treatment planning initiated
2022 (18 years)	Surgical phase	Extraction of tooth 16 and autotransplantation of tooth 28 using a CBCT-guided 3D replica	Successful transplantation and healing
2022 (18 years)	Orthodontic phase	Orthodontic appliances placed for mandibular space opening	Orthodontic treatment completed; prepared for surgical phase and future implant placement
2023 (19 years)	Implant placement	Guided flap surgery with Straumann BLT implants in sites 35 and 45	Stable osseointegration achieved
2024 (20 years)	Functional follow-up	3-year post-autotransplantation (from 2021) and 2-year post-implantation (from 2022); stable occlusion and satisfactory aesthetics	Stable occlusion and satisfactory aesthetics
2025 (21 years)	Micro-CT analysis	Extraction of teeth 18 and 48 for micro-CT evaluation using Rigaku CT Lab HX130	Confirmed therapy-related dental structural anomalies

BLT, bone level tapered; CSF, cerebrospinal fluid

### Diagnostic assessment

#### Diagnostic testing

Primary medical diagnoses included an undifferentiated small blue cell tumor of the right tibia with bone marrow involvement (2015, classified as stage IV disease on the basis of the presence of distant metastases) and relapsed B-cell precursor non-Hodgkin lymphoma with central nervous system involvement (2018, representing CNS relapse). Flow cytometry revealed a 71% blast population with B-precursor phenotype. Molecular testing confirmed IGH and TCR gene rearrangements consistent with B-precursor acute lymphoblastic leukemia.

Dental diagnoses included external root resorption of tooth 16 confirmed via periapical radiographs and CBCT, congenital agenesis of teeth 35 and 45 evident on panoramic radiographs, and developmental disturbances of third molars, including incomplete root formation and irregular morphology, confirmed post-extraction via micro-CT.

#### Diagnostic challenges

No specific diagnostic challenges were encountered. The dental anomalies were linked to the timing of oncologic therapy during active dental development.

#### Diagnosis

All dental findings were consistent with late effects of intensive chemotherapy and craniospinal radiotherapy administered during mixed dentition development.

### Therapeutic intervention

#### Types of therapeutic intervention

The patient underwent multidisciplinary oral rehabilitation to address dental anomalies resulting from the oncologic treatment.

Orthodontic appliances created adequate space in the mandibular arch for implant placement in sites 35 and 45. Due to progressive root resorption and structural instability, tooth 16 was extracted. Tooth 28 was selected as the donor for autotransplantation. A 3D-printed replica of tooth 28 was fabricated using CBCT data to pre-fit the recipient socket and minimize donor tooth handling (Fig. 3D) CBCT scans were acquired using i-CAT Next Generation, Kavo, Germany. A 3D-printed replica of tooth 28 was fabricated using CBCT data to pre-fit the recipient socket and minimize donor tooth handling. The digital workflow involved importing DICOM files from CBCT into planning software Materialise Mimics (version 24.0), where the replica was designed with precise dimensional specifications matching the donor tooth morphology [13].

#### Administration of therapeutic intervention

The autotransplantation procedure was performed atraumatically, with the transplanted tooth stabilized using 4–0 Vicryl sutures for two weeks. After sufficient healing and space creation, Straumann bone level tapered (BLT) implants (diameter 4.1 mm, length 10 mm) were placed at sites 35 and 45 under guided flap surgery using a CBCT-based 3D-printed surgical guide for accurate depth and angulation **(**Fig. 4C–E**)**. Digital implant planning was performed in Exoplan software using digital impressions (STL format) from Trios 5 intraoral scanner and DICOM files from CBCT. After confirmation of the plan by surgeon and prosthodontist, the surgical guide was designed and printed using Asiga 3D printer with Keyprint Keyguide resin at 50 micron resolution. Insertion torque ranged between 25–30 Ncm. Immediate healing abutments were placed, followed by a 3-month healing period before prosthetic restoration with zirconia crowns.

Teeth 18 and 48 were extracted 3 years post-treatment for structural analysis to evaluate therapy-related developmental changes. Molars were scanned using a 3D Micro X-Ray CT Scanner (CT Lab; Rigaku Corp., Tokyo, Japan). X-ray source voltage was set at 130 kV with tube current of 61 µA, focus set on S, and 0.1 mm copper filter applied. Scan acquisition settings included 14.52 mm field of view, short geometry, high-resolution scan mode, and high image resolution with 6-minute scan time. Before reconstruction, manual center adjustment and ring reduction filter were applied. Reconstructed CT scan images with slice thickness of 0.025 mm were exported as DICOM files and processed with Dragonfly software (version 2022.2.0.1399; Object Research Systems Inc., Montreal, QC, Canada).

#### Changes in therapeutic intervention

No modifications were made to the therapeutic intervention protocol during treatment.

### Follow-up diagnostic and other test results

- *Post-treatment tooth analysis: *Teeth 18 and 48 were extracted 3 years after final imaging and analyzed via micro-CT (Rigaku CT Lab HX130, Rigaku Corporation, Tokyo, Japan). Imaging revealed disorganized dentin-pulp morphology, incomplete root formation, and abnormal mineralization patterns (Fig. 6). These findings confirmed the hypothesis that chemotherapy and craniospinal radiotherapy during odontogenesis caused irreversible structural changes.
- *Follow-up protocol: *The autotransplantation follow-up schedule consisted of initial consultation and surgical planning, surgery, suture removal at 3 weeks, and radiographic evaluations at 3, 6, 12, and 24 months with periapical radiographs. Subsequent follow-up was conducted at 5 years in the absence of complications. Implant follow-up included evaluations at 3, 6, and 12 months post-placement, with annual reviews thereafter. No complications such as ankylosis, root resorption, or peri-implant pathology were observed during these visits.
- *Radiographic follow-up:* Sequential periapical and panoramic radiographs at 6-month intervals demonstrated successful integration of the autotransplanted tooth with continued root maturation. Implant sites showed stable bone levels with no signs of peri-implantitis or mechanical complications.

#### Intervention adherence and tolerability

Patient adherence was assessed through attendance at scheduled follow-up visits and compliance with postoperative care instructions. The patient attended all scheduled appointments and demonstrated excellent oral hygiene maintenance. Postoperative instructions were followed completely, including soft diet restrictions during initial healing phases and prescribed antibiotic regimens. Tolerability was excellent, with no reports of significant pain or discomfort beyond normal postoperative expectations.

#### Adverse and unanticipated events

No severe complications or unanticipated events were reported throughout the treatment period. Minor expected postoperative effects included transient swelling and mild discomfort following surgical procedures, which resolved within expected timeframes. The patient experienced no adverse reactions to local anesthesia, antibiotics, or analgesics. No signs of transplant rejection, implant failure, or infection were observed during the follow-up period.

From the perspective of the patient, 3 years after autotransplantation and 2 years after implant rehabilitation, high satisfaction was reported across multiple dimensions. Functionally, the autotransplanted tooth integrated completely and felt indistinguishable from her natural dentition during mastication. The aesthetics and function of implant crowns were excellent, allowing her to smile and eat without self-consciousness. She reported no sensitivity or discomfort with the autotransplanted tooth. However, she noted that hygiene maintenance beneath the implant crowns required more attention and specialized cleaning techniques compared with natural teeth. Despite this minor challenge, she valued the biological approach for preserving natural tooth structure and alveolar bone. She expressed no regrets over choosing autotransplantation and implants instead of conventional prosthetics such as bridges or removable partial dentures, emphasizing her preference for treatments that preserved as much natural tissue as possible. The patient appreciated the multidisciplinary team approach and felt well-informed throughout the treatment process.

## Discussion

This case demonstrates successful oral rehabilitation in a patient with dual malignancies and severe therapy-related dental anomalies using biological and digital approaches. Strengths include multidisciplinary planning, use of 3D technologies for precision, and objective assessment of treatment outcomes through micro-CT analysis. The approach preserved biological function while addressing aesthetic concerns through conservative methods rather than extraction-based rehabilitation. Limitations include the single-case design, which prevents generalization of outcomes. To our knowledge, this is the first report combining 3D-guided autotransplantation, implant rehabilitation, and micro-CT analysis in a survivor of dual pediatric malignancies. While implant rehabilitation is well documented in cancer survivors, autotransplantation in patients post-oncology has not been reported, nor has the integration of biological and digital approaches with structural assessment of therapy-related damage. The extended treatment timeline spanning multiple years required significant patient commitment and resources. Long-term follow-up beyond 3 years for autotransplantation outcomes remains unavailable. Additionally, the specific oncologic history may limit applicability to other patients after cancer treatment with different treatment protocols.

The patient’s oncologic history provides context for understanding treatment complexities and outcomes. The development of B-cell precursor non-Hodgkin lymphoma 3 years after rhabdomyosarcoma treatment represents a well-documented late effect of pediatric cancer therapy. Studies report that survivors of pediatric solid tumors have a four- to sixfold increased risk of developing secondary hematologic malignancies, with latency periods ranging from 2 to 10 years [14, 15]. This case aligns with established patterns of therapy-related secondary malignancies in childhood patients after cancer treatment.

Beyond secondary malignancy risk, childhood cancer treatment produces direct effects on developing dental structures. Chemotherapy and radiotherapy during odontogenesis cause well-documented dental anomalies. Literature reports that up to 80% of childhood patients after cancer treatment exhibit dental abnormalities when treated before the age of 10 years [3, 16]. The bilateral agenesis, root resorption, and third molar malformations observed in this case are consistent with systemic interference during mixed dentition development [1]. However, it should be noted that second premolar agenesis occurs in 3–4% of the general population, and while the timing suggests therapy-related effects, developmental variation may also contribute to this finding.

These dental anomalies necessitate biological treatment approaches that account for compromised healing capacity. Autotransplantation success rates reach 80–97.5% with appropriate case selection [17]. However, limited literature exists regarding autotransplantation in patients after cancer treatment, where healing capacity may be compromised. This case demonstrates feasibility in patients with compromised healing potential due to prior chemotherapy and radiotherapy. The successful 3-year outcome supports consideration of biological approaches in this population.

Digital planning technologies can optimize biological treatment outcomes in high-risk cases. CBCT-guided planning and 3D-printed surgical aids enhance autotransplantation outcomes by reducing extraoral time and improving precision [13, 18]. This case validates digital workflow integration in complex medical cases where surgical precision is critical.

When biological options are insufficient, implant-supported rehabilitation provides an alternative approach for patients after cancer treatment. Dental implant success in patients after cancer treatment varies on the basis of treatment history and bone quality. Studies report survival rates of 85–95% in patients post-oncology, with an implant failure rate of 4.5% observed in a retrospective study of 200 implants placed in 42 patients over a follow-up period of up to 8 years [19]. The successful 2-year implant outcomes in this case support guided placement protocols in carefully selected cases. The patient’s craniospinal radiotherapy dose of 18 Gy was substantially below the 50 Gy threshold associated with osteoradionecrosis risk [20]. Current guidelines indicate that ORN risk becomes significant when a cumulative dose to the jawbone exceeds 50 Gy [16, 20]. At the lower dose received by this patient, the risk of ORN from subsequent dental procedures was minimal, supporting the feasibility of autotransplantation and implant placement without the heightened precautions required for patients who received higher radiation doses to the head and neck region. The successful 2-year implant outcomes in this case support guided placement protocols in carefully selected cases.

This case illustrates that conservative, biologically driven approaches, such as autotransplantation, may offer feasible alternatives to conventional dental extractions in oncologic patients, opening new opportunities for functional rehabilitation and long-term preservation of oral structures.

## Conclusions

The biological approach combining autotransplantation and implant therapy proved successful despite the patient’s complex medical history. Micro-CT analysis provided objective evidence of therapy-related dental structural damage. The use of 3D technologies enhanced surgical precision and minimized complications. Patient-reported outcomes confirmed functional and aesthetic success, supporting the preference for conservative biological approaches with autotransplantation over extraction-based rehabilitation.

## Data Availability

All data generated or analyzed during this case are included in this published article.

## Abbreviations

ALL: Acute lymphoblastic leukemia
CBCT: Cone-beam computed tomography
CNS: Central nervous system
CSF: Cerebrospinal fluid
DNA: Deoxyribonucleic acid
FLAIR: Fluid-attenuated inversion recovery
IGH: Immunoglobulin heavy chain
MRI: Magnetic resonance imaging
NHL: Non-Hodgkin lymphoma
PET: Positron emission tomography
3D: Three-dimensional
μCT: Micro-computed tomography

## References

[1] Jaffe N, Toth BB, Hoar RE, Ried HL, Sullivan MP, McNeese MD. Dental and maxillofacial abnormalities in long-term survivors of childhood cancer: effects of treatment with chemotherapy and radiation to the head and neck. Pediatrics. 1984;73(6):816–23. 6728583

[2] da Fonseca MA. Dental care of the pediatric cancer patient. Pediatr Dent. 2004;26(1):53–7. 15080359

[3] Gawade PL, Hudson MM, Kaste SC, Neglia JP, Constine LS, Robison LL, *et al*. A systematic review of dental late effects in survivors of childhood cancer. Pediatr Blood Cancer. 2014;61(3):407–16. 24424790 10.1002/pbc.24842 PMC4281834

[4] Hölttä P, Alaluusua S, Saarinen-Pihkala UM, Wolf J, Nyström M, Hovi L. Long-term adverse effects on dentition in children with poor-risk neuroblastoma treated with high-dose chemotherapy and autologous stem cell transplantation with or without total body irradiation. Bone Marrow Transplant. 2002;29(2):121–7. 11850706 10.1038/sj.bmt.1703330

[5] Seremidi K, Gizani S, Dahllöf G, Barr-Agholme M, Kloukos D, Tsilingaridis G. Dental management of long-term childhood cancer survivors: a systematic review. Eur Arch Paediatr Dent. 2024;25(5):611–36. 38773051 10.1007/s40368-024-00896-5 PMC11442565

[6] Villa A, Akintoye SO. Dental management of patients who have undergone oral cancer therapy. Dent Clin North Am. 2018;62(1):131–42. 29126490 10.1016/j.cden.2017.08.010

[7] Hong CHL, Hu S, Haverman T, Stokman M, Napeñas JJ, den Braber JB, *et al*. A systematic review of dental disease management in cancer patients. Support Care Cancer. 2018;26(1):155–74. 28735355 10.1007/s00520-017-3829-y

[8] Levi LE, Lalla RV. Dental treatment planning for the patient with oral cancer. Dent Clin North Am. 2018;62(1):121–30. 29126489 10.1016/j.cden.2017.08.009

[9] Normando AGC, Pérez-de-Oliveira ME, Guerra ENS, Lopes MA, Rocha AC, Brandão TB, *et al*. To extract or not extract teeth prior to head and neck radiotherapy? A systematic review and meta-analysis. Support Care Cancer. 2022;30(11):8745–59. 35713725 10.1007/s00520-022-07215-y

[10] Peterson DE, Koyfman SA, Yarom N, Lynggaard CD, Ismaila N, Forner LE, *et al*. Prevention and management of osteoradionecrosis in patients with head and neck cancer treated with radiation therapy: ISOO-MASCC-ASCO guideline. J Clin Oncol. 2024;42(16):1975–96. 38691821 10.1200/JCO.23.02750

[11] Polder BJ, Van’t Hof MA, Van der Linden FPGM, Kuijpers-Jagtman AM. A meta-analysis of the prevalence of dental agenesis of permanent teeth. Community Dent Oral Epidemiol. 2004;32(3):217–26. 15151692 10.1111/j.1600-0528.2004.00158.x

[12] Gagnier JJ, Kienle G, Altman DG, Moher D, Sox H, Riley D. The CARE guidelines: consensus-based clinical case report guideline development. J Clin Epidemiol. 2014;67(1):46–51. 24035173 10.1016/j.jclinepi.2013.08.003

[13] Lejnieks M, Akota I, Jākobsone G, Neimane L, Radzins O, Uribe SE. Effect of 3D printed replicas on the duration of third molar autotransplantation surgery: a controlled clinical trial. Dent Traumatol. 2024;40(2):221–8. 37915275 10.1111/edt.12905

[14] Friedman DL, Whitton J, Leisenring W, Mertens AC, Hammond S, Stovall M, *et al*. Subsequent neoplasms in 5-year survivors of childhood cancer: the Childhood Cancer Survivor Study. J Natl Cancer Inst. 2010;102(14):1083–95. 20634481 10.1093/jnci/djq238 PMC2907408

[15] Armstrong GT, Liu Q, Yasui Y, Huang S, Ness KK, Leisenring W, *et al*. Long-term outcomes among adult survivors of childhood central nervous system malignancies in the Childhood Cancer Survivor Study. J Natl Cancer Inst. 2009;101(13):946–58. 19535780 10.1093/jnci/djp148 PMC2704230

[16] Hölttä P, Alaluusua S, Saarinen-Pihkala UM, Peltola J, Hovi L. Agenesis and microdontia of permanent teeth as late adverse effects after stem cell transplantation in young children. Cancer. 2005;103(1):181–90. 15540242 10.1002/cncr.20762

[17] Tan BL, Tong HJ, Narashimhan S, Banihani A, Nazzal H, Duggal MS. Tooth autotransplantation: an umbrella review. Dent Traumatol. 2023;39(Suppl 1):2–29. 36898857 10.1111/edt.12836

[18] Lejnieks M, Akota I, Jākobsone G, Neimane L, Uribe SE. Clinical efficacy of CBCT and 3D-printed replicas in molar autotransplantation: a controlled clinical trial. Dental Traumatol. 2025;41(2):161–70. 10.1111/edt.13012 PMC11907221 39506454

[19] Brauner E, Valentini V, Romeo U, Cantore M, Laudoni F, Rajabtork Zadeh O, *et al*. Dental implant failure risk in post oncological patients, a retrospective study and Sapienza head and neck unit decisional protocol-7 years of follow-up. Diagnostics. 2022;12(8):1863. 36010214 10.3390/diagnostics12081863 PMC9406984

[20] Saunders D, Koyfman SA, Ismaila N, Futran ND, Mowery YM, Watson E, Yang DH, Peterson DE. Prevention and management of osteoradionecrosis in patients with head and neck cancer treated with radiation therapy: ISOO-MASCC-ASCO guideline clinical insights. JCO Oncol Pract. 2024;20:1571–74. 10.1200/OP.24.00182 38691818

